# Elastic Scattering Spectroscopy (ESS): an Instrument-Concept for Dynamics of Complex (Bio-) Systems From Elastic Neutron Scattering

**DOI:** 10.1038/srep34266

**Published:** 2016-10-05

**Authors:** Antonio Benedetto, Gordon J. Kearley

**Affiliations:** 1 School of Physics, University College Dublin, Dublin, Ireland; 2 Laboratory for Neutron Scattering, Paul Scherrer Institut, Villigen, Switzerland; 3 School of Materials Science and Engineering, UNSW Australia, Sydney, NSW 2052, Australia

## Abstract

A new type of neutron-scattering spectroscopy is presented that is designed specifically to measure dynamics in bio-systems that are difficult to obtain in any other way. The temporal information is largely model-free and is analogous to relaxation processes measured with dielectric spectroscopy, but provides additional spacial and geometric aspects of the underlying dynamics. Numerical simulations of the basic instrument design show the neutron beam can be highly focussed, giving efficiency gains that enable the use of small samples. Although we concentrate on continuous neutron sources, the extension to pulsed neutron sources is proposed, both requiring minimal data-treatment and being broadly analogous with dielectric spectroscopy, they will open the study of dynamics to new areas of biophysics.

Whilst structure is clearly a key ingredient in understanding protein function, dynamics is at least as important in understanding mechanisms^1^,^2^,^3^,^4^. For example: the open-close dynamics that allow myoglobin to work efficiently^5^,^6^,^7^,^8^; the activation of proteins and enzymes by hydration water^1^,^2^,^3^,^4^,^9^,^10^,^11^,^12^; and, more generally, the dynamics of hydration water for life and in bio-structures^13^,^14^,^15^,^16^,^17^,^18^,^19^,^20^,^21^,^22^,^23^,^24^,^25^. Nevertheless, despite at least 3 decades of biophysical research in the field and the development of powerful experimental techniques, such as time-resolved X-ray diffraction^5^,^6^,^7^,^8^,^26^,^27^,^28^,^29^, the detailed connection between dynamics and (protein) biochemical function is still poorly understood. As a major example, the nature and the meaning of the “protein dynamical transition” is still, after almost 30 years, under debate^1^,^2^,^3^,^4^,^9^,^10^,^11^,^12^,^30^,^31^,^32^,^33^,^34^,^35^,^36^,^37^,^38^. There is an apparent lack of an adequate tool for measuring dynamics in complex bio-systems. In principle, inelastic neutron scattering should provide the solution because it has great sensitivity to protein motions, and those of hydration water, both via the displacements of their H-atoms that scatter neutrons incoherently with high probability. Unlike other spectroscopies, neutrons probe spacial and temporal aspects of the dynamics making it considerably easier to determine which species is actually being observed, and the nature of the motion. Because deuterium has a much lower scattering probability, the neutron signal can be further clarified by selectively “deuterating-out” components of the sample^1^,^3^,^4^,^9^,^19^,^22^,^35^,^37^,^38^. To date however, the technique has been hindered by the need for rather large samples, a perceived need to understand the neutron scattering process, and the scarcity of analogous lab-based spectroscopies. In the present publication we present a new type of neutron spectroscopy that overcomes all three limitations and should make a major contribution to the understanding of dynamics in complex bio-systems.

The current publication proposes a new neutron instrument specifically for the emerging biophysics community, which rather than adapting an existing design, starts from the principle requirements for measuring dynamics in biosystems. For brevity we concentrate on two basic designs that require no development of new instrumental elements: one for a continuous neutron source and one for a pulsed source. The use of new optical components and different designs would be straightforward. Dynamics is usually obtained via energy spectra, but our instrument determines the overall relaxation of the system under study by measuring the neutron-scattering amplitude as a function of the upper limit in relaxation rate (essentially resolution). The theoretical background for this neutron measurement has already been published in Rev. Sci. Instr.^39^,^40^, and here we design an instrument to measure the inflection of the function directly. This function is easily accessed experimentally from the apparent elastic-scattering intensity as a function of elastic-energy resolution, so we denote the instrument as an “elastic scattering spectrometer” (ESS). Neutron spectroscopies can miss potential applications because they lack laboratory analogues, but dielectric spectroscopy does measure a signal that is similar to ESS, but without the “neutron advantages” given above.

At present the main neutron-scattering technique used for the dynamics of bio-systems is quasi elastic neutron scattering (QENS), which has been largely developed by the physics/chemistry community to follow relaxation dynamics of much simpler atomic or small molecular species in solids and liquids^41^,^42^,^43^,^44^. The measured QENS signal is the Fourier transform of the space/time trajectories of each H-atom in the system, summed over all of these^41^. The experimental data are fitted with models for each type of H-atom in the system, taking its trajectory as convolution of all the translational, rotational and vibrational dynamics in which it participates. Clearly, this type of analysis is intractable for biosystems (and many complex systems) that contain a vast number of different H-atom environments, but nevertheless, it has been undertaken even though it usually only provides some approximation to an overall relaxation process. We will show that ESS can transform the measurement of this overall relaxation dynamics to a simple direct process.

We establish that the proposed instrument would work, and also that for measuring overall relaxation dynamics of biosystems it is both easier and more efficient than established QENS techniques. We demonstrate this by performing numerical simulations on the instrument design using a standard neutron-instrument simulation package McStas^45^.

## Background

The theoretical background to our instrument-concept has been known for some time, although not yet applied as we propose. Recently, the theoretical background was adapted towards new approaches^39^,^40^, where the general approach was called Resolution Elastic Neutron Scattering (RENS), our instrument, ESS, being a specific case of this. The starting point is:


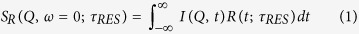


Eq. (1) shows that there will be an inflection point in the plot of the elastic intensity versus the instrumental energy-resolution when the energy resolution matches the overall system relaxation-time (SM Fig. 1 and SM Fig. 2). In complex and biological systems it is difficult to extract much more than a single relaxation processes from standard QENS, and it is not surprising to find that it is then more efficient to measure inflection point in Eq. (1). In QENS we collect intensity as a function of the energy transfer with a fixed energy resolution, whilst with RENS we collect intensity as a function of the energy resolution at fixed (zero) energy transfer. In principle these are equivalent, but because QENS instruments are required to measure complex profiles at many momentum-transfers, they suffer from a number of constraints that can be removed or relaxed in ESS.

We can show the useful range of energy resolution for RENS by considering three different domains (SM Fig. 1): (a) τ_RES _≫ τ, (b) τ_RES_ ≈ τ, and (c) τ_RES_ ≪ τ. The measured elastic intensity is the time integral of the product of the intermediate scattering function with the resolution function (Equation (1)), so it depends on the τ_RES_. In case (a) this integral is the area covered by the system intermediate scattering function, and the instrument is able to resolve the system dynamics. Whilst τ_RES _≫ τ nothing changes, and because we see essentially the integral of the system intermediate scattering function, this denoted the “system domain”. QENS is usually forced to measure too much of this domain. In contrast, in case (c) the time integral corresponds to the area of the resolution function, the system dynamics being too slow to be resolved. Again, whilst τ_RES_ ≪ τ the time integral will be τ_RES_ independent, and correspondingly this is denoted the “instrument domain”. The important case for RENS is (b) where τ_RES_ ≈ τ, which is denoted as the “resonance domain”. Here the time integral (i.e. the measured elastic intensity) is τ_RES_ dependent, where small changes in τ_RES_ give significant changes in the measured elastic intensity, and all measured data relate to the system relaxation dynamics. The system relaxation time is obtained directly from the inflection point in the plot of intensity vs energy resolution corresponding to the system relaxation time (SM Fig. 2).

**Figure 1:**
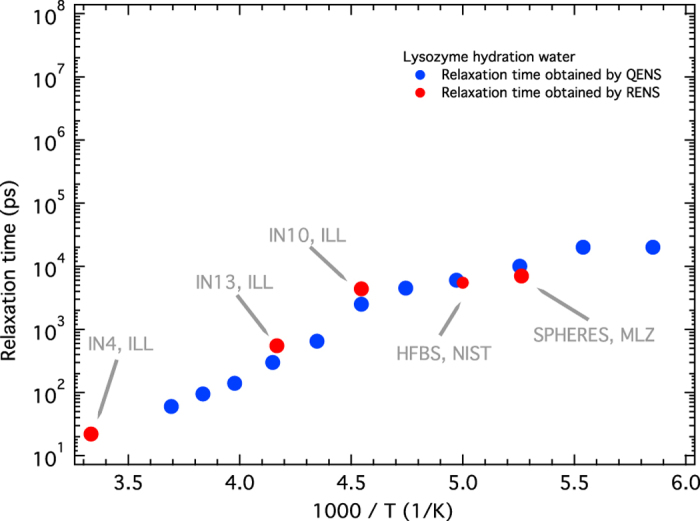
Relaxation time versus temperature of protein hydration water in Lysozyme measured by neutron scattering. The relaxation time obtained with the standard quasi-elastic neutron scattering protocol by Chen and co-workers^3^ (blue dots), and the one obtained by RENS protocol^37^,^38^ (red dots) are shown. The RENS data have been obtained by collecting the elastic neutron-scattering intensity with five different neutron spectrometers working at 5 different energy resolution: 0.65 μeV for SPHERES at MLZ, 0.8 μeV for HFBS at NIST, 1 μeV for IN10, 8 μeV for IN13, and 200 μeV for IN4 at ILL. In the current work we present an instrument-concept able to collect RENS-like data, and give then direct access to the system relaxation time. The main target will be complex (bio-) systems.

The RENS protocol can be currently accessed using backscattering spectrometers at a single incident-energy, so consequently several instruments are required, as has illustrated in Fig. 1. Alternatively, with time-of-flight (either direct or inverted) only the elastic-peak can be analysed using different resolutions, but this is extremely inefficient because most of the spectral time-frame is wasted. We will discuss how a quasi-backscattering approach can be used, but in which the energy-width of the incident beam can be tailored to scan the resolution over a wide range. In addition to simplicity and improved efficiency, there is the potential to focus on small samples (~1 mm^3^), which is a considerable advantage for biological materials that are usually only available in limited quantities. The concept would be compact, allow table-top like sample environments, and would not require a beam-end position.

## Results: Elastic scattering spectroscopy (ESS)

The instrument layout is broadly analogous to backscattering machines, in which neutrons scattered by the samples are analysed at a single energy, with the spectrum being scanned by modulating the energy of neutrons arriving at the sample. In the ESS instrument it is the energy resolution that is modulated, this being possible because we require only very modest momentum-transfer (Q) resolution compared with a general purpose QENS application.

### The primary spectrometer: a spatial-focussing time-defocussing monochromator

**Figure 2:**
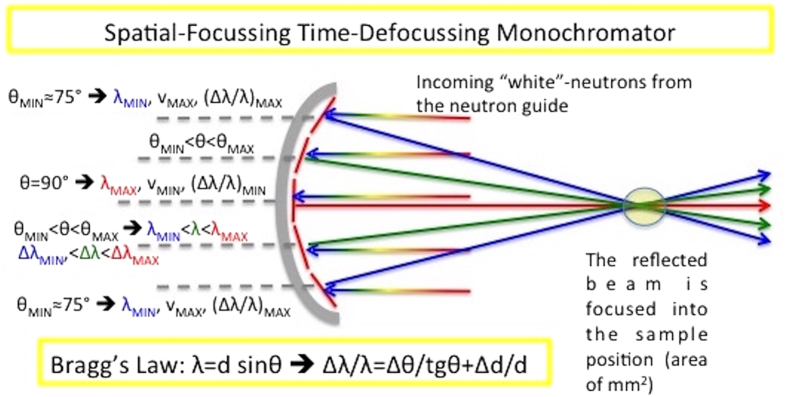
The primary spectrometer concept. The different colours represent different wavelength, which also have different energy resolutions.

The first element is a focussing monochromator, which is acceptable because of the relaxed Q-resolution. This element selects neutrons from the guide and focuses them onto the sample, achieving not only a concentrated beam current on the sample, but also bringing different energy resolutions onto the sample. This is illustrated schematically in Fig. 2, where for simplicity we have considered a single curvature of the monochromator, described by 5 equal flat crystals. The central crystal is in backscattering geometry (Bragg-angle θ ≈ 90), and provides the longest wavelength λ_MAX_ and the best resolution (Δλ/λ)_MIN_. As we move outwards through the crystals θ decreases, θ_MIN_ < θ < θ_MAX_, until θ_MIN_ for the outer crystal, giving the worst resolution (Δλ/λ)_MAX_. The five crystals thus provide three different energy resolutions, simultaneously, and the challenge is to disentangle these.

### The secondary spectrometer: Time-of-Flight (TOF) and Constant Wavelength (CW) options

The primary spectrometer (Fig. 2) provides the spread of energy resolutions, which are then distributed by the elastic and inelastic scattering of the sample, and we need to work back to the energy resolution. This process is tractable because we are only concerned with elastic scattering and is easily achieved using an almost standard crystal-analyser backscattering set-up. In this arrangement neutrons coming from the guide are reflected by the curved monochromator into the sample, then scattered by the sample to reach a set of analyser crystals (matched with the middle crystal of the curved monochromator) in backscattering geometry. Only the neutrons with the energy λ_MAX_ and distribution/resolution (Δλ/λ)_MIN_ will be backscattered by the analysers to the detectors. As a result the elastic intensity only (as a function of Q) is collected, but now this can be collected as a function of the energy resolution. In the simplest case (discussed in detail here) this is achieved by varying the curvature of monochromator, providing an overall resolution for each curvature. One limitation immediately arises. Although the contributions to the overall energy resolution are perfectly matched between the analyser crystals and the central monochromator crystal, there is no straightforward way in which this match can be maintained at the diffraction angles of other crystals. Whilst this is not conceptually satisfying, it is unlikely to be a significant problem in practice, because as the energy resolution worsens the net count-rate increases substantially, with a corresponding decrease in the count-time required.

**Figure 3:**
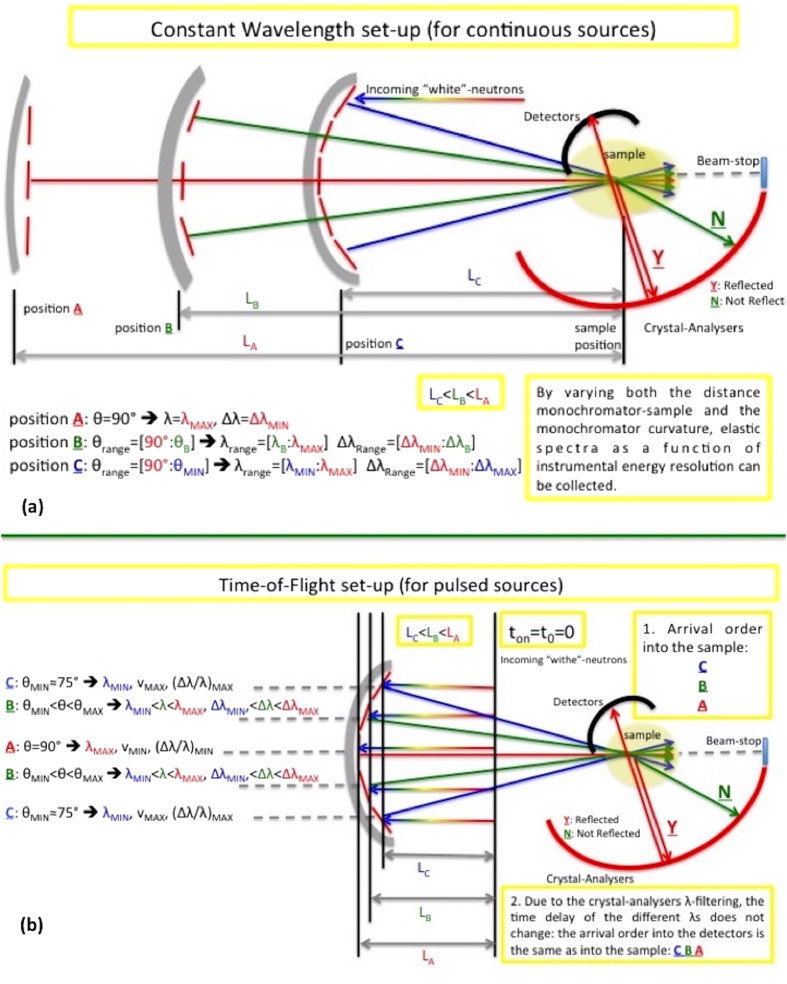
Instrument layout. (**a**) The layout for the CW instrument-concept suitable for continuous sources; and (**b**) the layout for the TOF instrument-concept suitable for pulsed sources. The different colours represent different wavelengths, which also have different energy resolutions, different velocities and, in turn, different TOF.

This layout for the CW option is presented schematically in Fig. 3a, where scanning the energy resolution by moving and refocussing the monochromator, maintains the small sample-size requirement. The high-resolution set-up is achieved by moving the monochromator as far as possible from the sample (2.9 m in the McStas simulations) to reduce its curvature (position A of Fig. 3a), providing a set-up that is similar to a high-resolution backscattering spectrometer. In contrast, the worst energy resolution is achieved by bringing the monochromator as close to the sample as possible (position C of Fig. 3a) and intermediate resolutions corresponding to intermediate positions such as position B in Fig. 3a. In all cases only scattered neutrons with λ_MAX_ will match the analyser Bragg condition, and inelastic processes at the sample may shift the incident energy to λ_MAX_. Effectively, the overall energy-resolution is largely determined by the monochromator energy-distribution. The output is the elastic intensity as a function of both the energy resolution and Q, although the Q-resolution will be poor due to the focussing.

The adaptation to pulsed sources by using the TOF option is illustrated in Fig. 3b. This option has the advantage that the energy resolution scans can be made without moving the whole monochromator. The spatially focussing monochromator is time-defocusing: neutrons from the middle monochromator-crystal have the lowest velocity and the longest trajectory to the sample, velocities getting higher and trajectories getting shorter as we move outwards across the monochromator. By using TOF at the detectors we can back-track to the monochromator element that provided the incident neutron, and hence energy resolution. Whilst the time-defocussing at the curved monochromator is helpful, it is not adequate to provide sufficient separation of the different resolutions, unless either the primary flight-path is very long, or sharp pulses can be generated (e.g. counter-rotating disc-choppers at a CW source). It is more practical to increase the spread of flight-paths by either increasing the distance between the monochromator segments along the incident-beam direction, or better, to stack the different monochromators in such the manner as proposed for the instrument Musical^46^. Separations of up to 500 microseconds can easily be achieved in this way, more details being provided in the supplementary material.

Generally speaking, it is possible to design other approaches able to give access to the elastic intensity as a function of the energy resolution, e.g. short time-frame QENS and other multi-pulse TOF approaches. Initial consideration of these is that they would be more complex and expensive to construct, and that the simplicity of our design is the key to attracting our target user (interdisciplinary) community.

### Validation of the spectroscopy

**Figure 4:**
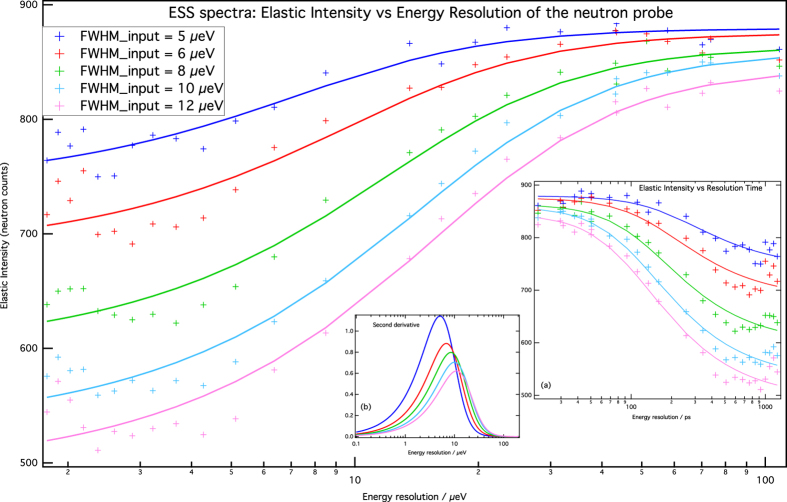
The ESS spectra for the CW version: the elastic scattered intensity as a function of the energy resolution for five samples with different quasi elastic widths (relaxation times) as indicated on the plot. The curves are the best fits of sigmoidal functions (Eq. 11 in ref. 39) of the experimental points. Inset: (**a**) the ESS spectra as a function of the resolution time in picosecond; (**b**) the second derivative of the ESS spectra showing a maximum at the energy value that matches the system relaxation time.

The feasibility of our instrument concept has been demonstrated by in-silico experiments using McStas, which is a widely accepted package for simulating neutron instrument and optics^45^. Details of the simulations are in the method section. Although we have made simulations for both CW and TOF versions in the energy-resolution range of 1.8 to 110 μeV (i.e. accessing two order of magnitude in the time space domain), we have concentrated more on CW, and that is what we present here (for TOF see Supplementary materials). We present computed ESS spectra for samples with 5 different system relaxation-times (Fig. 4).

Even a casual comparison of these curves with those in SM Fig. 2 reveals good agreement with theory: these show a sigmoidal behaviour with the inflection points of each curve matching the system relaxation times (see Table 1).

The table includes the system relaxation-times obtained by standard QENS, and the relative errors for both ESS and QENS. For details on the comparison refer to the method section. We took the most pessimistic option for ESS, by giving QENS advantages such as beam-focussing and optimised spectra range/resolution, that in practice it cannot achieve. The comparison of ESS to QENS (for a single relaxation process) was actually motivated as a validation of our simulation, knowing that the two methods should be essentially equivalent under these rather unrealistic conditions, not as a “competition”. As anticipated, there is no significant difference between the errors in the relaxation time that we determine for equivalent ESS and QENS simulations. This comparison was achieved by transforming the signal from the sample in the simulation to either QENS or ESS.

The gains for ESS are not in the number of neutrons, but in the beam-focussing, concentrating on the region in which the relaxation-dynamics of the system resonates with the energy resolution of the instrument, and in the model-free analysis. However, where detailed analysis of the QENS profile is required, then QENS is obviously the better choice.

## Discussion

In the above we establish the validity and the feasibility of our instrument concept, and here we will establish the practical advantages of ESS in the measurement of complex and biological systems. Firstly, the overall relaxation-time is determined from QENS by analysis of the quasi-elastic profile in terms of likely functions. Even starting from the assumption that only an overall relaxation-time via QENS, the data need to be deconvoluted from the experimental resolution and transformed from the energy to the time domain. Otherwise, if a priori generic functions are chosen there is often contention because a number of models frequently give similar fits. It is generally accepted that if the fitted function contains more than about 5 independent adjustable parameters, it is more realistic to accept an overall relaxation time which we believe is more efficiently obtained using ESS.

Sample size is a key issue for neutron scattering in biophysics, and we must optimise the phase-space resolution and range in both energy and momentum transfer. For energy, this optimisation is made naturally by the RENS theory that ESS uses. QENS instruments aim to accommodate some coherent scattering with a consequent demand for good Q-resolution, but ESS is entirely concerned with incoherent scattering and can afford spatial focussing down to ~1 mm^2^ by sacrificing unwanted Q-resolution. The sample size will be comparable with typical biochemistry methods, and together with the interest in collecting the solely elastic intensity make it possible to measure biosystems in physiological environments, e.g. low concentration of protein in a water buffer, something that is almost impossible with QENS.

The ESS spectrum would normally be collected as a function of wave vector Q (as in QENS), to obtain some spatial analysis of the relaxation dynamics. Typically, this would be restricted to a distance or volume and either confined or long-range diffusive motion. Where separable relaxation processes are present, theses will show up as different inflection points in the ESS spectrum. Whilst it is tempting to claim that this will provide direct and model-free access to several processes, the analysis is far less developed than for QENS, and our primary aim remains overall relaxation-dynamics in complex systems. For more information see supplementary materials.

The instrument simplicity and smaller samples, make a more “table-top” or multi-technique approach to sample environment possible. Although a cold-neutron source would always be required this means that our instrument could not only be well suited to major neutron facilities, but also to smaller sources, especially the radio frequency quadrupole (RFQ) based neutron generators that are currently being developed.

### Conclusions and outlook

We have presented a concept for a new type of neutron spectroscopy aimed mainly at measurement of relaxation times in complex (bio-) systems. This type of instrument collects the solely elastic intensity as a function of the energy resolution, from which the inflection points give a direct measure of the system relaxation times, without need for the a-priori models used in QENS. We considered two slightly different instruments, one designed for continuous sources, and one for pulsed sources. We have validated these with McStas simulations. In our simulation we were able to cover more than 2 orders of magnitude in energy resolution (1–100 μeV), to focus the neutron beam on a sample size of 1 mm^2^, and to achieve the same relative error as standard QENS, even when QENS was given impracticable advantages.

By focussing we achieve smaller sample sizes than with standard QENS, which offers considerable advantages where sample availability is a problem. Recent developments in optical components for neutron scattering offer opportunities to improve considerably on our initial design, particularly improvements in spatial focussing and TOF defocussing. We would hope that this spectrometer would inspire new users, particularly from the biology community, to neutron scattering, and instrumentalists to develop the idea further.

Finally, it should be possible to devise a highly compact “table-top” spectrometer that would be fundamentally different from any current design; this will open the possibility to perform neutron scattering experiments that could drive a “scientific revolution” in the studies of the relaxation dynamics in complex (bio-) systems.

## Method: ESS validation and comparison with QENS by McSats

To establish the feasibility of our instrument concept we simulated the basic set-up with McStas. We deal primarily with the CW version and use only standard components, accepting that for TOF and specialised focusing elements would offer significant improvements. We also tested the TOF version, which even in a basic set-up, was able to access the same energy resolution range of the CW version.

The CW and TOF options sketched in Fig. 3a,b, respectively, were developed to a more practicable definition for the McStas simulation. The McStas code of the simulation is available as supplementary material, and the final instrument parameters are collected in SM Table 1. In the following we begin with the CW version, and then return to the TOF version.

### The CW version

The simulation provided promising results for both a standard vanadium sample that was used for calibration, and for a simple sample with a single relaxation process. The scans of elastic intensity as a function of the energy resolution for the sample show clear inflection points at the energy resolution corresponding to the system relaxation times, as predicted by the theory. Although the range of energy resolutions could probably be improved by deflecting the beam from the monochromator away from the guide (as in some existing backscattering spectrometers) our simple instrument was still able to access an energy-resolution range from around 1 to more than 100 μeV using sample-monochromator distance from 3 m to 10 cm, respectively(SM Fig. 3).

Each ESS curve has 25 points, corresponding to the 25 positions (and curvatures) of the monochromator, at distances between 0.11 and 2.9 m (SM Fig. 3), the corresponding energy resolutions being 110 μeV to 1.8 μeV, respectively. To access this resolution range, we made use of the option to change the monochromator angle, but limiting this to 5.6 degrees, the correspondence between monochromator angle and resolution being illustrated in the inset of SM Fig. 3.

Initially, a simple calibration of the instrument was made with vanadium as a sample. Because vanadium is a purely incoherent scatterer, with essentially no inelastic scattering in our energy region, only those neutrons originating from central crystal of the monochromator are detected after the analyser. SM Fig. 4 illustrates the range of wavelengths leaving the monochromator (column 1), then the range scattered by the vanadium sample (column 2), and finally the range that passed the analyser to reach the detector (column 3) using energy-resolution set-ups of 1.8 μeV, 5 μeV, 16 μeV.

The energy resolution of a set-up depends on the neutron-energy distribution from the curved monochromator (column one of SM Fig. 4). For analysis of our simulations the resolution-value of each set-up (each monochromator-sample distance) was obtained from the FWHM of a Gaussian fitted to the profile in column 1 (SM Fig. 4). We also tested a graphical method, but this gave no significant difference from fitting with Gaussian functions. The obvious deviation from a Gaussian profile will shift the FWHM from the true value, causing some systematic error in the abscissa of the final plot. A numerical approach to obtaining the effective width of the distribution would avoid these errors.

For a “real” sample we use a model system with 80% of the scattering distributed within a Lorentzian function of adjustable width representing a specific relaxation time. SM Fig. 4 shows the signal at different parts of the spectrometer for FWHM = 8 μeV using energy-resolution set-ups of 1.8 μeV, 5 μeV, 16 μeV. The columns are: range of wavelengths leaving the monochromator (column 1); the range scattered by the “real” sample (column 2); range that passed the analyser to reach the detector (column 3). We use the data in the first column to obtain the resolution of the set-up. The analogy with QENS is recognisable after scattering by the sample (column 2) where particularly for the highest resolution (top row) the classic elastic and quasi elastic components are clear. Finally, after the analyser we see the same profile for each resolution, but its integral depends on the resolution from the monochromator. The ESS spectrum is actually proportional to this integral.

For each ESS scan we use 25 resolution values, corresponding to 25 monochromator-to-sample distances. The resulting spectra are normalised by an incident-beam monitor (integral from the monochromator), and also by the identical scan using vanadium as sample. Each final ESS spectrum is given by:


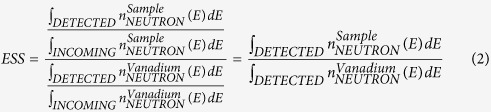


We made full simulations for five different system-relaxation times (i.e. with FWHM = 5, 6, 8, 10, and 12 μeV), and the final results are reported in Fig. 4.

### ESS vs QENS

QENS is the method of choice where analytical models can be used successfully to determine relaxation and molecular processes, but where we need to establish an overall relaxation time in complex (bio-) systems, ESS is a more efficient approach. We can make a reasonable comparison between the two methods for relaxation time only by using data based on SM Fig. 4, in which the second column is the QENS signal. Use of these data gives QENS some instrumental advantages that can only be realized with the ESS set-up. These are: spatial focussing; the choice the most efficient resolution for QENS (τ_RES_ = 3τ); and finally the energy range is matched to the relaxation process under study.

The number of incident neutrons for the two methods, QENS and ESS was equal: each of the 25 ESS points uses N incoming neutrons, whilst for QENS a single run using 25N neutrons was made. QENS spectra were fitted with a Lorentzian function to describe the quasi-elastic component of the system and a delta function for its elastic component both convoluted with a Gaussian for taking in proper account the instrumental energy resolution; the curves in SM Fig. 5 are the fitting curves. The ESS spectra were fitted using a sigmoidal-like function, as the one reported in Eq. (11) of ref. 39, to determine the inflection point (i.e. model-free); the curves in Fig. 4 are the fitting curves. The results of the fits are reported in Table 1.

### Validation of the TOF version

The TOF version has been also validated by McStas simulations where the goal was to separate the elastic peaks originating from each crystal segment of the monochromator by a time delay longer than 200 microseconds for an average incident flight-path of 30 m. The slowest peak comes from the central element and has the best resolution, and the fastest peak comes from the crystal element at the edge of the monochromator with the worst energy resolution. In our basic set-up, we separate the elastic peaks between each monochromator element by about 500 microseconds (SM Fig. 6), which requires a total monochromator separation of 2.9 m to achieve the same resolution range of the CW set-up, i.e. from 1.8 μeV to 110 μeV. The different distributions are shown in SM Fig. 6. The spatial arrangement of these crystal elements can be chosen to tune the delay between peaks, and an arrangement in which different monochromators are stacked (similar to Musical^46^) would maximise the use of the incident beam, but would be complex to construct. By integrating each elastic peak in the TOF spectrum, we obtain a very similar ESS spectrum to that shown in Fig. 4.

### Determination of more relaxation processes and Q-analysis

We emphasise that our focus is on the ability of ESS to determine directly the system relaxation-time without a-priori functions and fittings. This is ideally suited where a single overall relaxation process is of interest. If more relaxation processes are present this will alter the ESS response because each of the relaxation processes in the system will generate a corresponding inflection point in the ESS spectrum.

As with QENS, if the relaxation times are sufficiently distinct the processes can be analysed separately. For idealised spectrometers there is probably little difference between the ability of QENS and ESS to separate these processes. However, QENS instruments have been optimised for this purpose over many decades and it would make little sense to optimise ESS for the same purpose and correspondingly we aim at maximum efficiency for one, or maybe two overall relaxation processes.

ESS spectra can be collected as a function of the exchange wave-vector Q. This has two components: the wavelengths and the analysers angles with respect to the beam incident on the sample. The wavelengths from the different monochromator elements vary within a small range (SM Fig. 6a), so the average Q at a given analyser angle for each resolution is virtually the same. In addition, the angular divergence is large so the overall Q-resolution is rather modest, so highly accurate average values are unlikely to be useful. In practice, the Q-variation associated with the dynamical processes of interest here is a smooth function, so relaxed Q-resolution is not a problem.

## Additional Information

**How to cite this article**: Benedetto, A. and Kearley, G. J. Elastic Scattering Spectroscopy (ESS): an Instrument-Concept for Dynamics of Complex (Bio-) Systems From Elastic Neutron Scattering. *Sci. Rep.* **6**, 34266; doi: 10.1038/srep34266 (2016).

## Supplementary Material

Supplementary Information

Supplementary Information

## Figures and Tables

**Table 1:** Relaxation times and their relative errors obtained by ESS and QENS.

τ_INPUT/μeV	τ_ESS/μeV	τ_QENS/μeV	Δτ/τ ESS %	Δτ/τ QENS %
5	4,918	5,250	−1,637	4,990
6	6,492	6,102	8,200	1,708
8	8,585	8,522	7,306	6,522
10	10,136	10,404	1,355	4,038
12	11,240	12,874	−6,338	7,284
		<Δτ/τ>/%	4,967	4,908

ESS adopts a model-free approach aimed to extract the abscissa of inflection points, whereas QENS needs an a priori model that here was the same one used as input in the simulations, something that is not possible in real experiments where any model can be proposed a priori. The error on the relative error is 1.2%, so ESS and QENS have the same ability but QENS was given all the advantages, which is not usually realistic in practice.
