# Supplementary material for: Elastic Scattering Spectroscopy (ESS): an Instrument-Concept for Dynamics of Complex (Bio-) Systems From Elastic Neutron Scattering
Source: Sci Rep. 2016 Oct 5;6:34266. doi: 10.1038/srep34266 (PMC5050422; doi:10.1038/srep34266)
Supplement: Supplementary Information [file srep34266-s1.pdf]

Elastic Scattering Spectroscopy (ESS): an Instrument-Concept for Dynamics of Complex (Bio-) Systems From Elastic Neutron Scattering

Antonio Benedetto[1,2], and Gordon J Kearley[3]*

[1]*School of Physics, University College Dublin, Dublin, Ireland*

[2]*Laboratory for Neutron Scattering, Paul Scherrer Institut, Villigen, Switzerland*

[3]*School of Materials Science and Engineering, UNSW Australia, Sydney, NSW 2052, Australia*

**Corresponding author: antonio.benedetto@psi.ch*

Supplementary information

$$S_R(Q,\omega=0;\tau_{RES}) = \int_{-\infty}^{\infty} I(Q,t)R(t;\tau_{RES})dt$$

---*I(t;τ)*: *system function* at fixed value of *τ=1.5×10*^{3} *ps*;

---*R(t;τ*$_{RES}$*)*: three resolution functions with τ$_{RES}$ *=10*^{5} *ps*, *2000 ps* and *200 ps*;

---*S*$_R$*(Q,ω=0;τ*$_{RES}$*)*: *Measured* elastic scattering laws

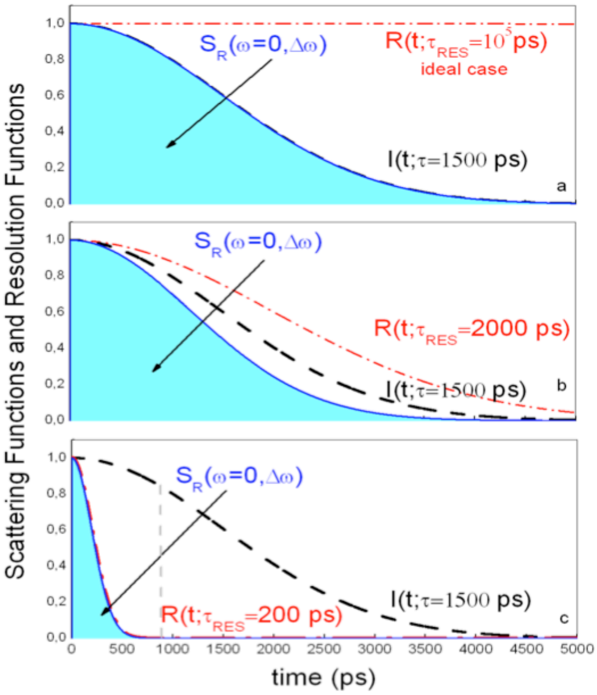

Three regimes/domains:

(a) ***τ$_{RES}$>>τ*** -> "System domain": $S_R=S_{system}$ and ***τ$_{RES}$* independent**. QENS regime.

(b) ***τ$_{RES}$≈τ*** -> "Resonance domain": S_R ***τ$_{RES}$* dependent**. RENS regime.

(c) ***τ$_{RES}$<<τ*** -> "Instrument domain": S_R=R and ***τ$_{RES}$* independent**. We are measuring the resolutio function (e.g. QENS @ 1K).

SM Fig. 1 – A summary of the theoretical background.

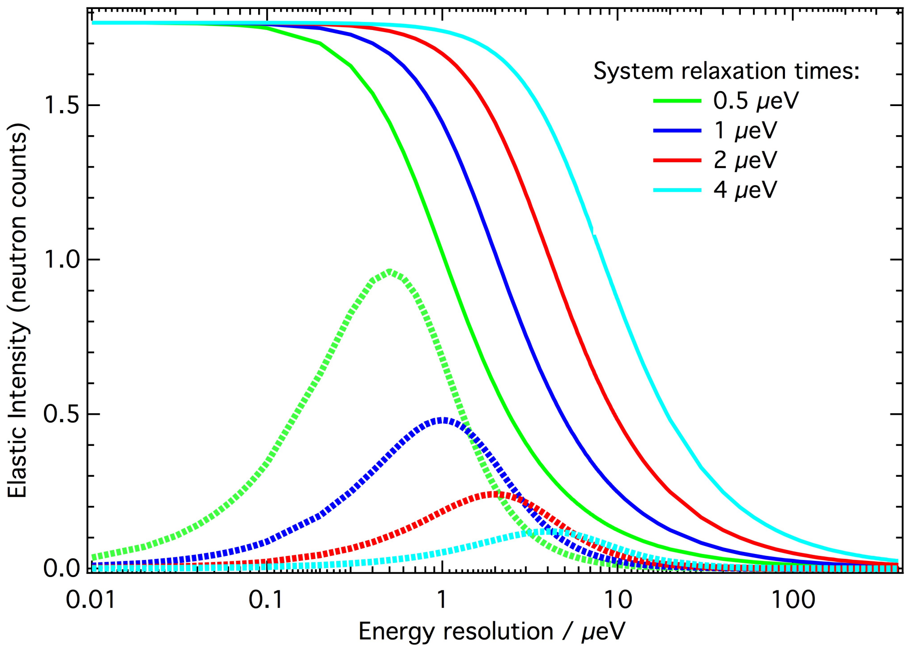

SM Fig. 2 – Theoretical curves elastic intensity as a function of the energy resolution (Eq. 11 of Ref. [39]). The inflection points occur where the energy resolution is equivalent to the system relaxation times, confirmed also by the second derivative (dashed lines) showing a maximum at the energy resolution values that match the system relaxation times.

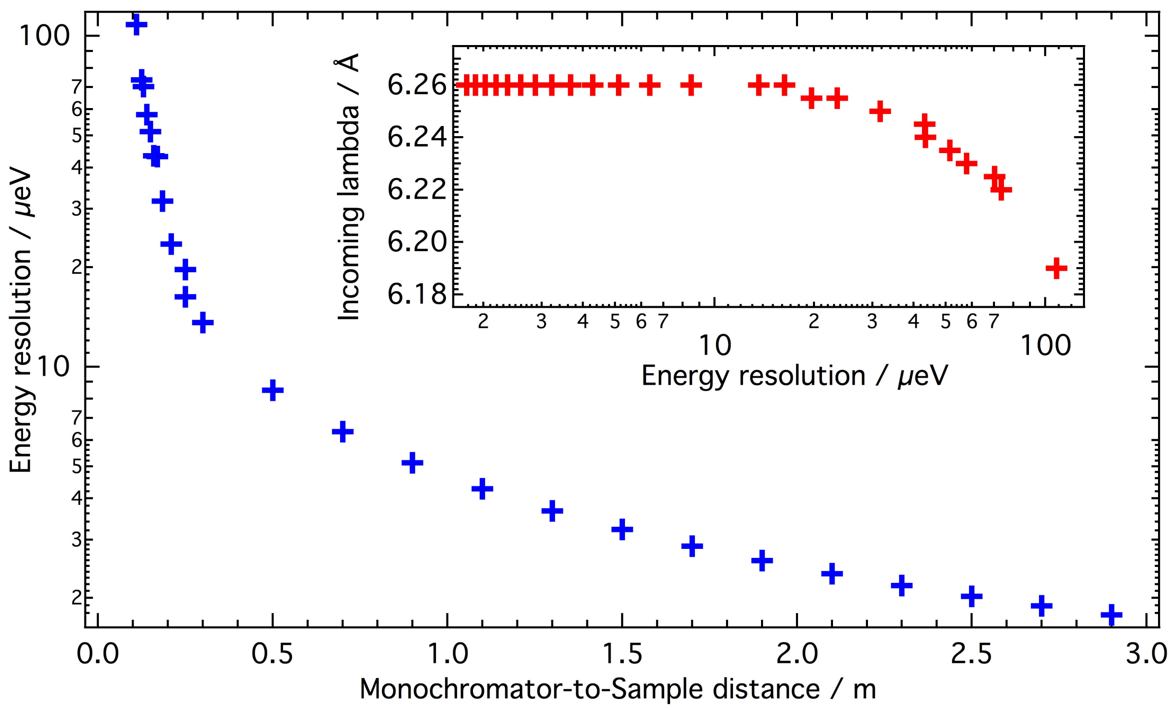

SM Fig. 3 – Energy resolution versus monochromator-sample distance. The 25 different resolution values correspond to the different monochromator-to-sample distances. Inset: incoming lambda versus instrumental energy resolution.

**Source**	
Width of the source/ m	0.05
Height of the source / m	0.05
Width divergence / deg	1
Height divergence / deg	1
Mean wavelength of neutrons / Å	*see SM Fig. 3*
Wavelength half spread of neutrons / Å	0.2
**Guide**	
Width at the guide entry	0.05
Height at the guide entry	0.05
Width at the guide exit	0.04
Height at the guide exit	0.04
m-value of material	1
Low-angle reflectivity	0.995
Critical scattering vector / Å^-1	0.0217
Slope of reflectivity / Å	5.76
Width of supermirror cut-off / Å^-1	0.003
**Monochromator**	
Distance to the guide exit / m	0.75
Width of an individual slab / m	0.0001
Height of an individual slab / m	0.0001
Gap between adjacent slabs / m	0.0000125
Number of slabs vertical	400
Number of slabs horizontal	400
Vertical mosaic (FWHM)	10
Horizontal mosaic (FWHM)	10
Maximum reflectivity	0.99
Monochromator d-spacing / Å	3.1355 (Si, 111)
Radius of vertical focussing / m	2***dms**
Radius of horizontal focussing / m	2***dms**
**Sample**	
distance to monochromator: **dms**	*see SM Fig. 3*
sample geometry	annular
Thickness / m	0.005
Outer radius / m	0.008
Height / m	0.01
**Analyzers**	
Distance to sample / m	1
Width of an individual slab / m	0.01
Height of an individual slab / m	0.01
Gap between adjacent slabs / m	0.005
Number of slabs vertical	40
Number of slabs horizontal	40
Vertical mosaic (FWHM)	10
Horizontal mosaic (FWHM)	10
Maximum reflectivity	0.99
Monochromator d-spacing / Å	3.1355 (Si, 111)
Radius of vertical focussing / m	1
Radius of horizontal focussing / m	1
**Detectors**	
distance to analyzers / m	1

SM Table 1 – Parameters of the simulated instrument. “dms" is the distance monochromator-to-sample, which varies between 0.11 and 2.9 m (SM Fig. 3); FWHM stays for full weight at half maximum. The layout of the instrument is the one in Fig. 3a. The calculations were carried out at double precision.

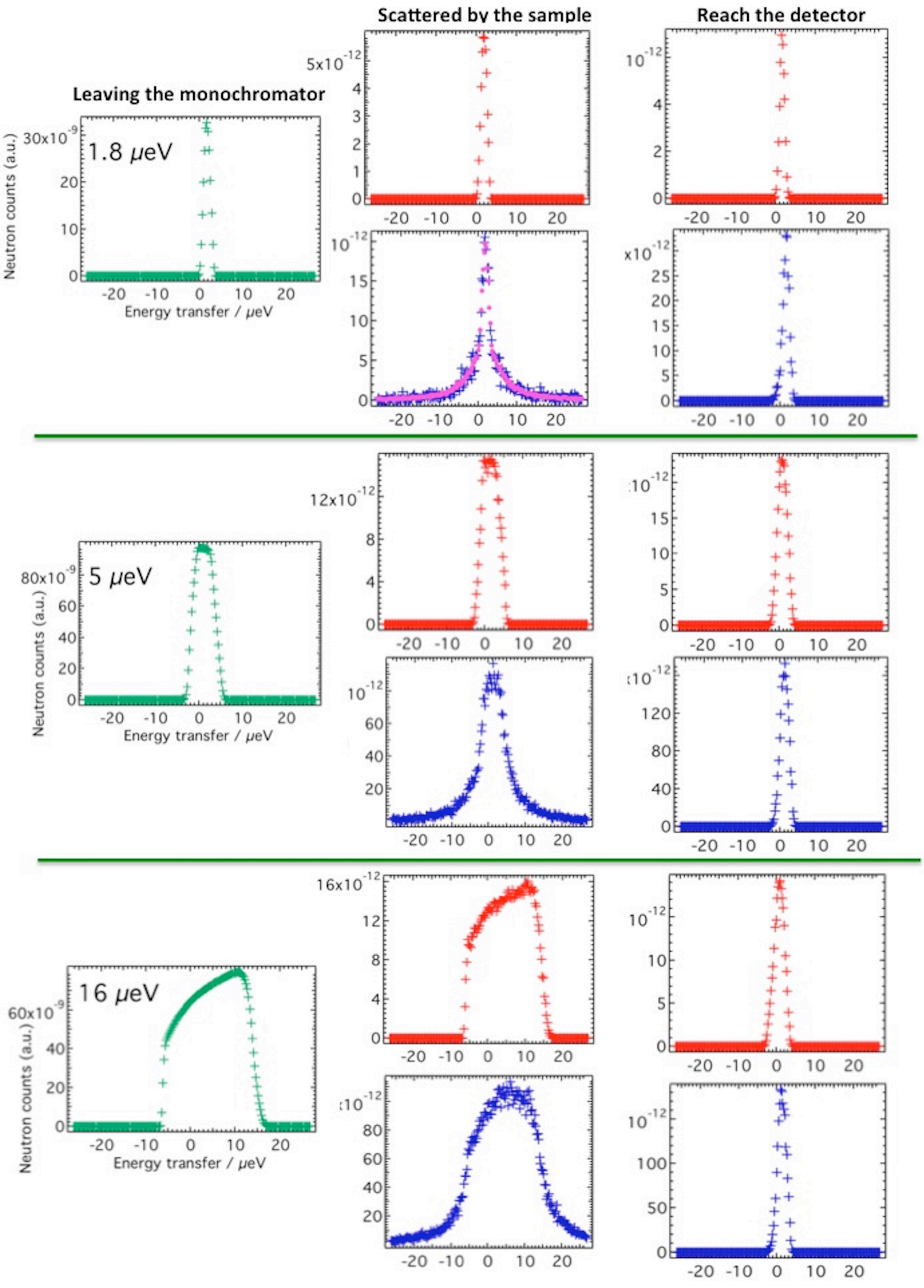

SM Fig. 4 – Energy distribution for both vanadium (red) and sample (blue) from the curved monochromator (column one), scattered by the sample/vanadium (column two), and after the analyser at the detectors (column three) at energy resolution: 1.8μeV (top row), 5μeV (middle row), and 16μeV (bottom row). The “real” sample is a standard incoherent scatterer with 20% elastic (delta function) and 80% quasi-elastic (Lorentzian function, FWHM=8μeV) components. The energy distribution from the monochromator is the same for both vanadium and the sample (green). Second column, second line: In pink an example of QENS spectra with the better statistics (25xN incoming neutrons) used to compare QENS and ESS is superimposed on the standard statistic case for ESS (N incoming neutron).

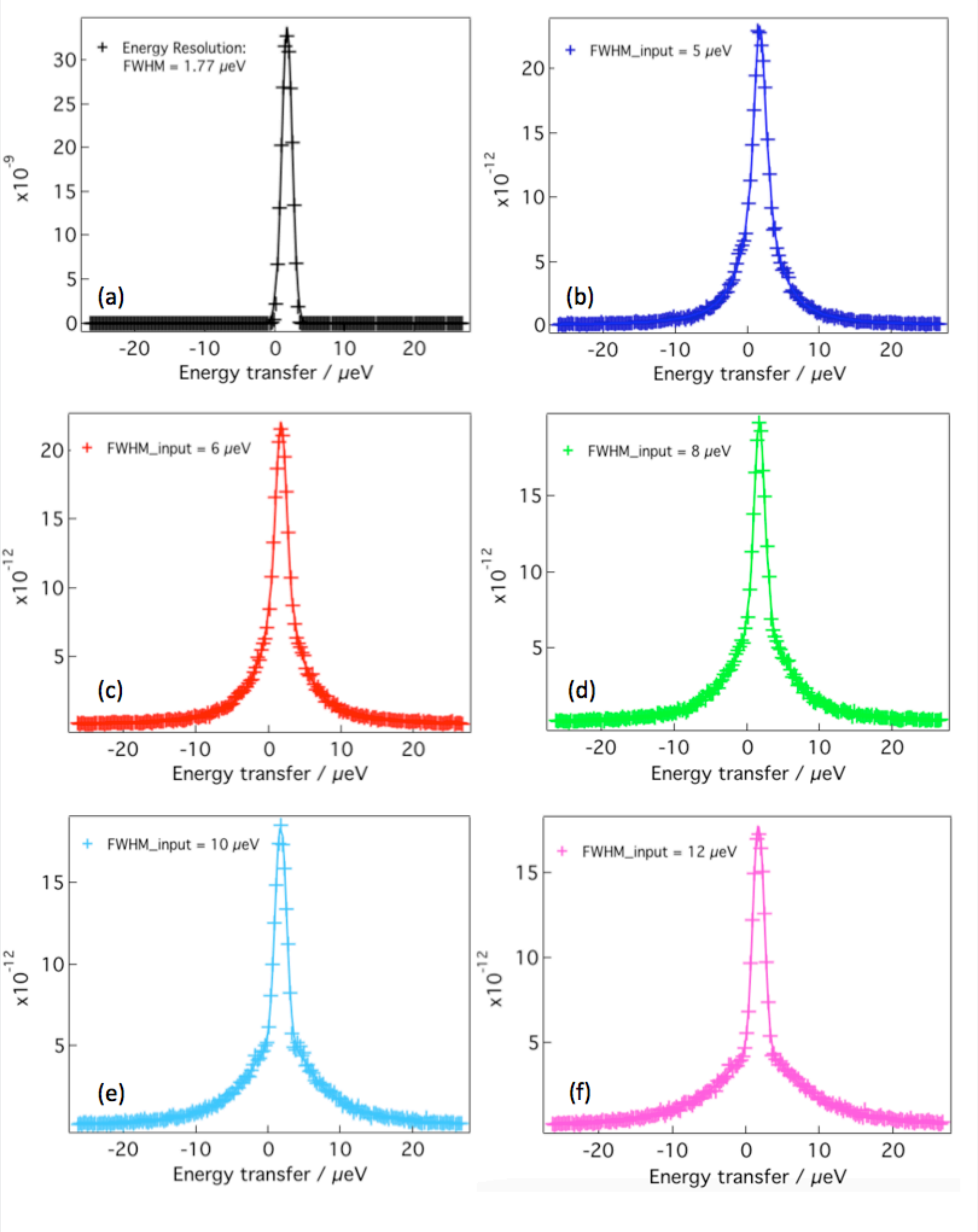

SM Fig. 5 – QENS spectra (dots) and best fits (curves) with improved statistics (25xN incoming neutrons) used to compare ESS and QENS, as in Table 1. The set of QENS curves is for the best accessible energy resolution (a), i.e. 1.77μeV, at different relaxation times of the system (i.e. FWHM of the Lorentzian): from 5μeV in (b) to 12μeV in (f). In all cases the QENS is in an ideal configuration ($\tau_{RES} \approx 3\tau$) to probe the dynamics of the system. Even in this case QENS is not better than ESS (Table 1 in the main text). QENS is not usually in its ideal set-up, so ESS is a better way for system dynamics.

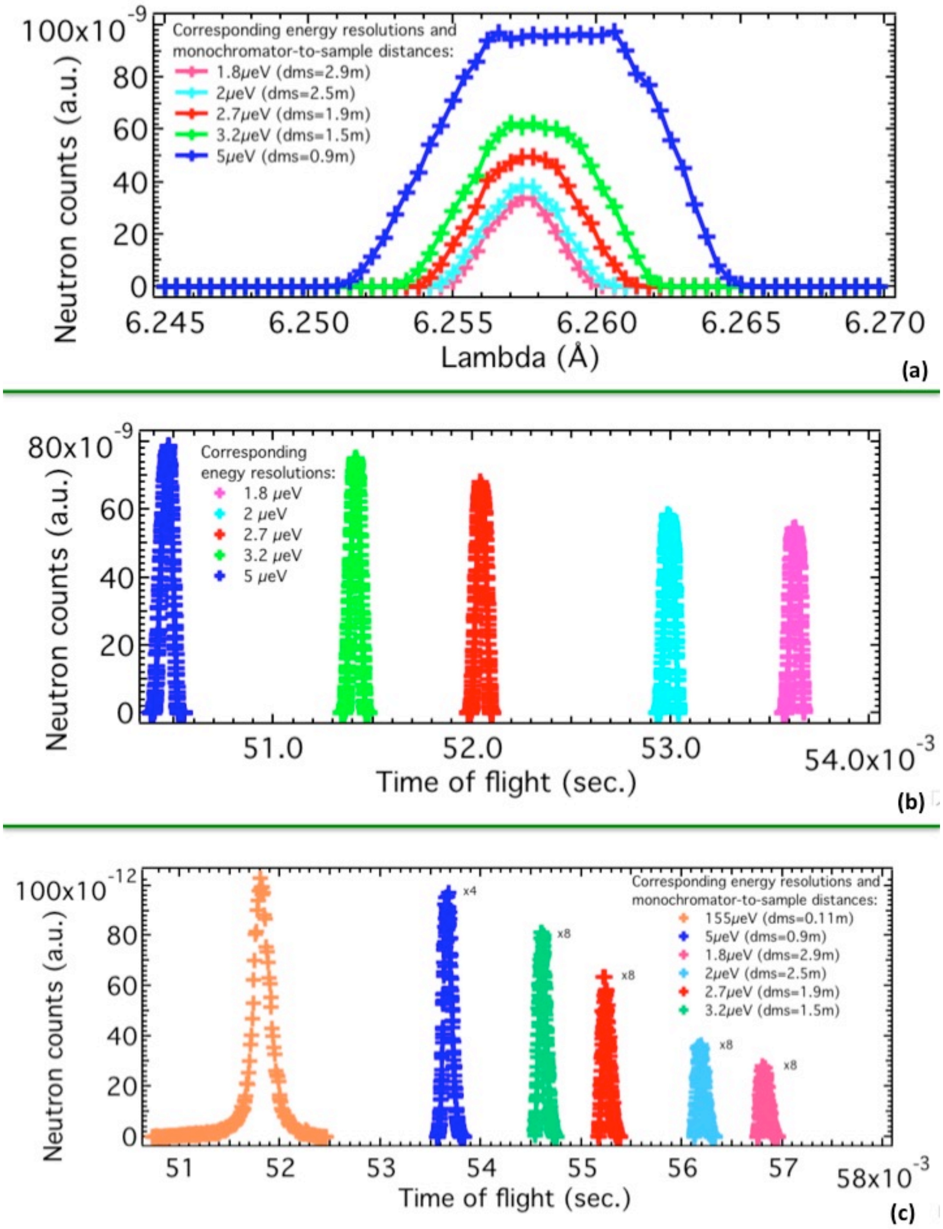

SM Fig. 6 – The TOF version: results from McStas simulations. Each of the different crystal elements of our spatial-focussing time-defocusing monochromator of Fig. 2 (main text) reflects into the sample neutrons having slightly different lambdas but all distributed around about 6.26 Å, with, in turn, different energy resolutions related to the width of each lambda-distribution (a). This gives access to two orders of magnitude in energy resolution, i.e. 1.8 to 110 μeV. By displacing the crystal elements by 2.9m along the direction of the incoming beam, the time-defocusing ability of the monochromator was tuned to separate by about 500 microseconds each lambda distribution at the sample position (b). The lambda and TOF distributions refer to the neutrons coming into the sample (they have been collected at the sample position). The scattering process with the sample and the reflection by the analysers is the same as for the CW version: only the sharp high resolution lambda distribution from the central crystal element will be reflected by the analysers and then collected by the detectors, but at different TOF depending on the energy resolution and the monochromator crystal element they came from (c). The longest flight path was 34.9m, whereas the shortest 32.11m. The time delay between the different lambda-distributions does not change and allow indeed to back-track at the detector position the original energy resolution giving access to the ESS spectra, as in Fig. 4.
